# Social capital and use of assisted reproductive technology in young couples: Ecological study using application information for government subsidies in Japan

**DOI:** 10.1016/j.ssmph.2021.100995

**Published:** 2021-12-06

**Authors:** Seung Chik Jwa, Osamu Ishihara, Akira Kuwahara, Kazuki Saito, Hidekazu Saito, Yukihiro Terada, Yasuki Kobayashi, Eri Maeda

**Affiliations:** a Department of Obstetrics and Gynecology, Saitama Medical University, 38 Morohongo, Moroyama, Saitama, 350-0495, Japan; b Department of Obstetrics and Gynecology, Graduate School of Biomedical Sciences, Tokushima University, 3-18-15, Kuramoto-cho, Tokushima, 770-8503, Japan; c Department of Pediatrics, Perinatal, and Maternal Medicine (Ibaraki), Graduate School, Tokyo Medical and Dental University, 1-5-45, Yushima, Bunkyo-ku, Tokyo, 113-8510, Japan; d Umegaoka Women's Clinic, 1-33-3, Umegaoka, Setagaya-ku, Tokyo, 154-0022, Japan; e Department of Obstetrics and Gynecology, Graduate School of Medicine, Akita University, 1-1-1, Hondo, Akita, 010-8543, Japan; f Department of Public Health, Graduate School of Medicine, The University of Tokyo, 7-3-1, Hongo, Bunkyo-ku, Tokyo, 113-0033, Japan; g Department of Environmental Health Science and Public Health, Akita University Graduate School of Medicine, 1-1-1, Hondo, Akita, 010-8543, Japan

**Keywords:** Assisted reproductive technology, Income, Japan, Social capital, Use rate

## Abstract

**Background:**

Assisted reproductive technology (ART) is a globally established treatment; however, large disparities exist in ART use among young couples. We investigated regional-level factors associated with ART use in Japan.

**Methods:**

We calculated the use rate of ART using the number of women aged <35 years who applied for government subsidies in 2017; we divided that figure by the number of women aged 20–35 years in each prefecture. Prefectural-level average household income; social capital indicators including voting rate, volunteer rate, and move-in rate; and Gini coefficients as indicators of income inequality were linked to ART use, adjusting for prefectural size, the mean age of women at first marriage, number of ART facilities, and additional prefectural subsidies.

**Results:**

The rate of ART use (per 10,000 women) varied significantly from 22.0 to 58.8 across Japan's 47 prefectures. Multivariate analysis demonstrated that the use rate increased by 0.048 (95% confidence interval [CI], 0.007 to 0.088) for each 10,000-yen increase in average household income and 1.5 (95% CI, 0.65 to 2.3) for each 1% increase in volunteer rate. Conversely, the use rate decreased by 18.4 (95% CI, −28.6 to −8.1) for each 1% increase in the move-in rate. There was no significant association between ART use and income inequality.

**Conclusion:**

Although we cannot infer causal relationships, the findings suggest that improving financial access and enhancing social capital may increase access to ART. Further research, particularly multilevel analysis using individual data, is required to confirm these findings.

## Highlights

• There was significant variation in ART use in women aged <35 years across Japan.
• This study is the first to show positive associations between prefectural-level social capital and ART use.
• Prefectural-level mean household income was positively associated with ART use.
• Though we cannot infer causal relationships, improving financial access and enhancing social capital may increase ART use.

## 1 Introduction

Infertility affects 8%–12% of reproductive-age couples worldwide (Boivin et al., 2007; Datta et al., 2016; Ombelet et al., 2008) and causes substantial personal distress to millions of couples from all socioeconomic backgrounds. The treatment of infertility has been revolutionized over the last four decades, primarily because assisted reproductive technology (ART) has become a standard, wide-reaching treatment. According to a preliminary report from the International Committee for Monitoring Assisted Reproductive Technology, 1,882,018 treatment cycles were conducted in 78 countries in 2016; of these, over 328,885 were associated with deliveries (Adamson et al., 2020).

In 2016, Japan was the world's second-highest provider of ART treatment (447,763 cycles annually) after China (906,840 cycles annually). The number of treatment cycles in Japan was over double that in the United States, which was the third-highest ART provider (190,149 cycles) (Adamson et al., 2020). A major reason for the high number of ART cycles in Japan is infertility owing to advanced age. The latest report from the Japan Society of Obstetrics and Gynecology states that in 2018, the mean age of Japanese women receiving ART was 38.0 years, and 41.8% were aged over 40 years (Ishihara et al., 2021). ART treatment using donated oocytes or embryos is not legally permissible in Japan; thus, older women must undergo repeated treatment to achieve a live birth. By contrast, young couples in Japan tend to delay receiving ART owing to the high economic burden. Women's age is the most influential factor in determining the success of ART (Ben Messaoud et al., 2020). Therefore, identifying factors associated with ART use among young couples is necessary to establish effective public health strategies to improve use in this population.

There are several barriers to ART use, including a couple's ethnic or racial traits (Quinn & Fujimoto, 2016; Seifer et al., 2020), socioeconomic status (including income and educational level) (Chambers et al., 2013), and geographic access (Makuch et al., 2011). Most studies on barriers to ART have been conducted in Western countries; to the best of our knowledge, no research has been reported from Asia. Several other factors, such as social capital and income inequality, have been widely recognized as social determinants for various health outcomes and have recently received attention from governments (Centers for Disease Control and Prevention, 2013; Marmot et al., 2010; Marmot et al., 2012). Social capital comprises resources that an individual can access as a result of being part of a network or group (Bordieu, 1986), and has been defined in various ways(Rodgers et al., 2019). Putnam defines social capital as “features of social organization such as trust, norms and networks” (Putnam, 1994). To date, no studies have investigated the association between social capital and ART use. Therefore, the present study used prefectural-level data to investigate regional-level factors associated with use of ART by young Japanese couples.

## 2 Materials and methods

For this ecological study, we used application information for government subsidies for ART. In Japan, women aged under 40 years can be partly reimbursed for ART treatment fees up to six times, and women aged 40–42 years can receive such reimbursement up to three times. The reimbursement is subject to an annual upper household income limit of 7.3 million yen (approximately USD 64,000, 2021 exchange rate of $1 = 114 yen) per couple based on proof of earnings for prefectural tax or a taxation certificate for the previous year. Reimbursement applications are handled by local municipalities in the case of core cities (i.e., population >200,000) and ordinance-designated cities (i.e., population >500,000 and designated by the Japanese government under the Local Autonomy Law); otherwise, they are managed by prefectural governments. Patients must make an immediate out-of-pocket payment and then apply to the local municipality within the same fiscal year. A recently published nationwide survey investigating ART implementation across ART facilities reported that the median treatment cost for one treatment cycle (fresh cycles with subsequent embryo transfers) is 500,000 yen (4386 USD) (Ministry of Health Labour and Welfare, 2021).

We conducted a questionnaire survey in August 2018 of all cities and prefectural governments managing government subsidies for ART (48 core cities, 20 ordinance-designated cities, and all of Japan's 47 prefectural governments) to investigate the implementation of government subsidies. The response rate was 100%; no cities or governments that managed subsidies were excluded. The survey assessed the numbers of women who received subsidies and ART treatments in the 2017 fiscal year (April 2017 to March 2018) by age. We also assessed the following: whether the local government provided additional subsidies for infertility treatment; changes in age limits for reimbursement; changes in the number of reimbursed treatments; changes in the amount of reimbursement; changes in upper income limits for reimbursement; additional subsidies for non-ART treatment or infertility testing; and other factors. We defined additional subsidies at the prefectural level as positive if at least one local government within a prefecture offered such subsidies.

In total, 118,194 women aged under 35 years who received government subsidies during the 2017 fiscal year in Japan were included in the analysis. Using these data, we calculated the number of women in each prefecture who received government subsidies. We calculated the use rate of ART for women aged under 35 years per 10,000 women aged 20–35 years within each prefecture using data from the Ministry of Internal Affairs and Communications on the estimated population for each prefecture in October 2017 (Ministry of Internal Affairs and Communications, 2017a).

To assess income inequality, we used the Gini coefficient at the prefectural level. The Gini coefficient is defined mathematically as half of the absolute difference between two incomes selected randomly from a population normalized to the mean. The theoretical range of the Gini is between 0.0 (perfect equality) and 1.0 (perfect inequality); higher scores indicate greater inequality. We obtained the prefectural-level Gini coefficient using a national survey of family income and expenditure conducted in 2014 by the Ministry of Internal Affairs and Communications (Ministry of Internal Affairs and Communications, 2016b).

The intended level of analysis was macro (prefecture level). We selected voting, volunteering, and move-in rates as social capital indicators to evaluate different aspects of social capital. Voting rate was used as an indicator of civic engagement, and volunteer rate was used as an indicator of social cohesion. The move-in rate was selected as an indicator for networks of trust connecting individuals and groups; a high move-in rate indicates a weak network of trust. Thus, we assumed that voting rate would be a proxy for evaluating structural social capital, volunteer rate would be a proxy for cognitive social capital, and move-in rate would relate to linking social capital. We used the voting rate, volunteer rate, and move-in rate at the prefectural level. For voting rate, we used data for the Lower House election in 2017 (Ministry of Internal Affairs and Communications, 2017b). We determined the volunteer rate using the proportion of individuals who participated in volunteer activities in 2016 (Ministry of Internal Affairs and Communications, 2016c). We defined the move-in rate as the number of people moving to a target prefecture from another prefecture during 2016 divided by the number of people living in the target prefecture (Ministry of Internal Affairs and Communications, 2016a).

To assess other factors, we used the following variables: average annual income of households with more than two members at the prefectural level (Ministry of Internal Affairs and Communications, 2016b); population (Ministry of Internal Affairs and Communications, 2017a); area and number of physicians (Ministry of Health Labour and Welfare, 2016); and ART facilities (Ethics Committee of the Japan Society of Obstetrics and Gynecology, 2019) at the prefectural level as a proxy for accessibility. We used the mean age of women at first marriage (National Institute of Population and Social Security Research, 2017) as a proxy for need for infertility treatment.

### 2.1 Statistical analysis

We calculated Pearson correlation coefficients to examine the association between each indicator and ART use. Using univariate linear regression, we then investigated the association between each indicator and ART use. Population, area, number of physicians, and ART facilities were not normally distributed; thus, we used log transformations of these variables for analysis. We included all the variables in a multivariable model except for population and number of physicians; these two variables were associated with the number of ART facilities and so were excluded to avoid multicollinearity. Finally, to evaluate whether the number of women aged under 35 years who received government subsidies accurately reflected all women receiving ART, we asked the Saitama Prefectural Government to provide details of individual data for government subsidies for 2016 and 2017 (1928 women) and evaluated the distribution of total household income. We conducted all analyses using the STATA MP statistical package, version 16.0 (Stata, College Station, TX, USA) and Statistical Package for Biosciences Ver. 9.65 (Murata & Yano, 2002). We considered a two-tailed *P* value of <0.05 statistically significant. This study was approved by the institutional review board of Akita University (approval number, 1981; June 2018) and Saitama Medical University (approval number, 904; September 2019).

## 3 Results

**Fig. 1:**
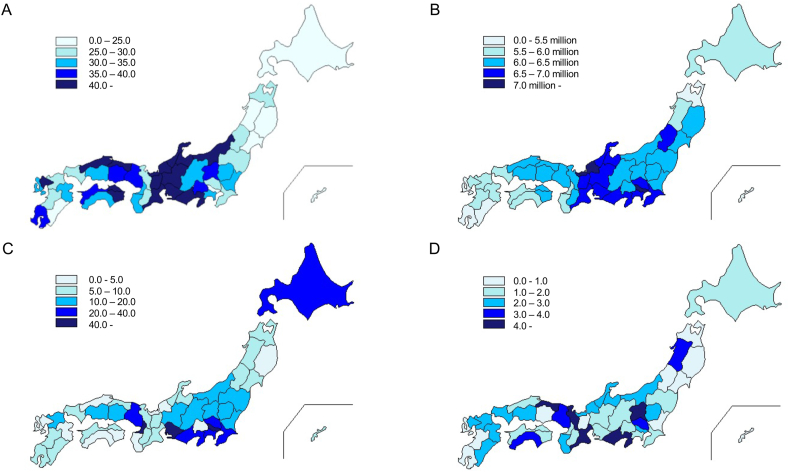
Prefectural distribution for (A) ART use for women aged under 35 years (per 10,000 women), (B) average household income (yen), (C) number of ART facilities, and (D) number of additional subsidies.

Table 1 shows the details of demographics, socioeconomic distribution (including social capital indicators), and government subsidies for ART at the prefectural level. In 2017, the mean number of women receiving government subsidies was 2515 (median, 1423; interquartile range [IQR], 831–2356); of these, the mean number of women aged under 35 years was 663 (median, 400; IQR, 230–685). Therefore, the mean proportion of women aged under 35 years among all women receiving subsidies was 26.7% (median, 26.7; IQR, 25.5–28.0). The mean use rate of ART for women aged under 35 years per 10,000 women was 35.2; it varied significantly from 22.0 to 58.8. Fig. 1 shows the prefectural distribution for ART use rate, average household income, number of ART facilities, and number of additional subsides. Regarding prefectural-level socioeconomic factors, the average household income was 6,170,000 yen (54,100 USD) (median, 6,120,000 yen [53,700 USD]; IQR, 5,781,000–6,538,000 yen [50,710–57,350 USD]). The mean Gini coefficient was 0.353 (median, 0.35; IQR, 0.342–0.363). The average volunteer rate was 27.9% (median, 27.8%; IQR, 25.2–31.6). The average move-in rate was 1.54% (median, 1.51%; IQR, 1.26%–1.71%). The average voting rate for the Lower House election was 58.7% (median, 58.7%; IQR, 55.5%–61.0%). In all, 36 prefectures (76.6%) provided additional subsidies for ART other than government subsidies. The types of subsidies most frequently provided were additional subsidies for non-ART treatment or infertility testing (23 prefectures, 48.9%), increases to the amount of reimbursement (20 prefectures, 42.6%), and increases to the number of reimbursements (11 prefectures, 23.4%).

**Table 1:** Demographics, socioeconomic distributions and governmental subsidies for ART of prefectures in Japan (N = 47).

	Mean (SD) or n (%)	Median (range)
Population (10,000 persons)	270.1 (274)	163 (57–1362.4)
Population of women at age 20–34 (10,000 persons)	20.4 (24.7)	11.8 (3.6–13.4)
Area (Km^2^)	8040 (11700)	6096 (1876–83,456)
Mean age at first marriage	29.1 (0.38)	29.1 (28.6–30.5)
Number of doctors	6797 (7583)	4081 (1805–44,136)
Number of ART facilities	13.1 (16.4)	7 (2–100)
Number of applications for governmental subsidies at prefecture		
Number of women receiving governmental subsidies in 2017	2515 (2830)	1423 (498–13835)
Number of women age <35 y receiving governmental subsidies^a^	663 (721)	400 (128–3241)
Proportion of women age <35 y out of total women receiving governmental subsidies	26.7 (2.2)	26.7 (19.1–31.2)
Utilization rate for women age less than 35 years (10,000 persons)	35.2 (6.1)	33.4 (22.0–58.8)
Household socio-demographic characteristics		
Average household income (10,000 JPY)	617 (56.6)	612 (470–769)
Average household size (person)^b^	2.4 (0.17)	2.5 (2.3–2.5)
Proportion of women graduated from university/college^c^	15.6 (3.6)	15.0 (13.3–18.6)
Proportion of foreign nationals^d^	1.4 (0.85)	1.2 (0.70–2.2)
Total fertility rate^e^	1.5 (0.14)	1.5 (1.4–1.6)
Socioeconomic factors		
Gini coefficient	0.35 (0.015)	0.35 (0.32–0.38)
Volunteer rate (%)	27.9 (3.5)	27.8 (20.6–33.9)
Move-in rate (%)	1.5 (0.39)	1.5 (0.91–3.0)
Voting rate in the Lower House election (%)	58.7 (4.1)	58.7 (50.2–68.6)
Additional subsidies for ART at prefecture (%)	36 (76.6)	–
Shifting age limitation for reimbursement (%)	2 (4.3)	–
Increase the number of reimbursement (%)	11 (23.4)	–
Increase the amount of reimbursement (%)	20 (42.6)	–
Shifting income limitation for reimbursement (%)	6 (12.8)	–
Additional subsidies for non-ART treatment or infertility testing (%)	23 (48.9)	–
Others (%)	10 (21.3)	–

ART, assisted reproductive technology; SD, standard deviation.

a In total, 118,194 women age less than 35 who received governmental subsidies during fiscal 2017 in Japan were included in the analysis.

b From census data 2015.

c From census data 2010.

d From Statistics of foreign residents in 2015.

e From the vital statistics 2017.

**Fig. 2:**
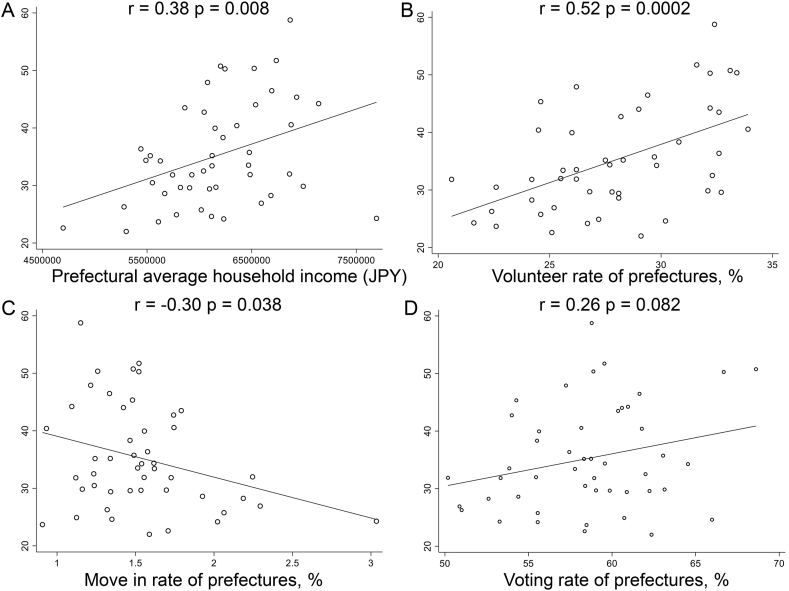
Correlations between prefectural average household income, social capital indicators, and ART use for women aged under 35 years (per 10,000 women). The vertical axis shows ART use for women aged under 35 years (per 10,000 women) and the horizontal axis shows prefectural average household income and social capital indicators.

Fig. 2 shows the correlations between average household income, social capital indicators, and ART use rate. We observed a significant positive correlation between average household income (*r* = 0.38, *P* = 0.008), volunteer rate (*r* = 0.52, *P* = 0.0002), and ART use rate for women aged under 35 years; there was a significant negative correlation between move-in rate and use rate (*r* = −0.30, *P* = 0.038). We found a non-significant correlation between voting rate and ART use (*r* = 0.26, *P* = 0.082).

Bivariate and multivariate coefficients for ART use for women aged under 35 years are shown in Table 2. The bivariate analysis showed that use rate had a significant negative association with log-transformed population and number of physicians. The multivariate analysis showed significant positive associations between average household income, volunteer rate, and use rate. Each 10,000-yen increase in average household income was associated with a 0.048 increase (95% confidence interval [CI], 0.007 to 0.088) in use rate. Similarly, each 1% increase in volunteer rate was associated with a significant increase in use rate (1.5; 95% CI, 0.65 to 2.3). However, each 1% increase in move-in rate was associated with an 18.4 (95% CI, −28.6 to −8.1) decrease in use rate. We observed no significant associations between Gini coefficient, additional subsidies for ART at the prefectural level, and use rate.

**Table 2:** Bivariate and multivariate coefficients for the use rate of ART for women age less than 35 years by prefecture.

	Bivariate analysis		Multivariate analysis	
	Coefficient (95% CI)^a^	p value	Coefficient (95% CI)^a^	p value
Log(population)	**−3.5 (-6.9 to -0.22)**	**0.037**	–	
Log(area (Km^2^))	−2.9 (−6.9 to 1.1)	0.15	**−7.1 (-11.0 to -3.1)**	**0.001**
Mean age at first marriage	−6.7 (−13.7 to 0.22)	0.06	2.0 (−7.3 to 11.4)	0.67
Log(number of doctors)	**−3.5 (-7.0 to -0.001)**	**0.05**	–	
Log(number of ART facilities)	−1.7 (−4.8 to 1.43)	0.28	1.9 (−1.5 to 5.2)	0.26
Mean household income (ref: 10,000 JPY increase)	**0.061 (0.016 to 0.11)**	**0.008**	**0.048 (0.007 to 0.088)**	**0.02**
Gini coefficient	−158 (−331 to 15.1)	0.07	59.4 (−78.9 to 198)	0.26
Volunteer rate (%)	**1.33 (0.67 to 1.99)**	**<0.001**	**1.5 (0.65 to 2.3)**	**0.001**
Move-in rate (%)	**−7.1 (-13.8 to -0.43)**	**0.038**	**−18.4 (−28.6 to −8.1)**	**0.001**
Voting rate in the Lower House election (%)	0.56 (−0.07 to 1.2)	0.08	−0.41 (−1.04 to 0.22)	0.20
Shifting age limitation for reimbursement	−2.5 (−15.8 to 10.9)	0.71	−2.5 (−14.3 to 9.3)	0.67
Increase the number of reimbursement	4.5 (−1.7 to 10.7)	0.15	1.7 (−3.0 to 6.5)	0.46
Increase amount of reimbursement	−0.25 (−5.7 to 5.2)	0.93	−0.25 (−4.4 to 3.9)	0.90
Shifting income limitation for reimbursement	6.1 (−1.7 to 14.0)	0.12	3.5 (−3.0 to 10.0)	0.28
Additional grant for non-ART treatment or infertility testing	1.0 (−4.4 to 6.4)	0.71	3.4 (−0.92 to 7.7)	0.12
Others	4.6 (−1.9 to 11.0)	0.16	−1.7 (−6.4 to 3.1)	0.47

ART, assisted reproductive technology; CI, confidence interval.

Bold values indicate p < 0.05.

a Coefficient for the ART use rate per 10,000 women according to the one-unit increase in each variable.

**Fig. 3:**
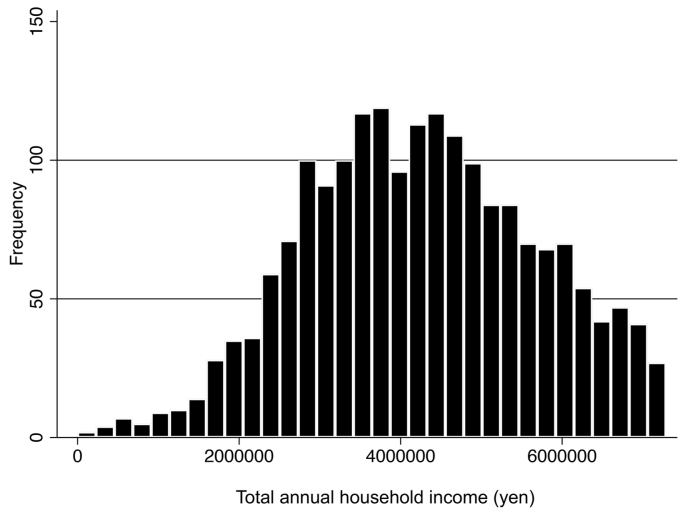
Distribution of total household income (JPY) for women aged under 35 years who received government subsidies from Saitama Prefectural Government (1928 women for 2016 and 2017). The vertical axis indicates frequencies and the horizontal axis indicates annual total household income (yen). The government subsidies have an annual upper household income limit of 7.3 million yen (approximately USD 64,000) per couple based on proof of earnings for prefectural tax or a taxation certificate.

Finally, Fig. 3 shows the distribution of total household income for women aged under 35 years who received government subsidies from Saitama Prefectural Government (1928 women for 2016 and 2017). The average total household income was 4,249,639 yen (37,278 USD) (standard deviation, 1,433,746 yen [12,577 USD]); almost the entire distribution was covered by the upper limit of overall household income.

## 4 Discussion

Our findings suggest that prefectural-level social capital may have a positive effect on ART use in young women. To the best of our knowledge, this is the first study to report an association between social capital and ART use.

Although no previous studies have investigated the association between social capital and ART use, it is possible to identify several reasons for this association. First, individuals with high social capital are more likely to receive medical care; many studies have investigated the relationship between social capital and healthcare-seeking behavior and outcomes (Chuang et al., 2015; Derose & Varda, 2009; Honda et al., 2018; Inoue et al., 2020; Rodgers et al., 2019; Santoso et al., 2020). A recent survey of 8770 employees aged 18–70 years found that both men and women with low workplace social capital had a significantly higher risk of not seeking medical care than those with high workplace social capital group (Inoue et al., 2020). Similarly, several studies have investigated the relationship between social capital and health during pregnancy (Agampodi et al., 2017; Story, 2014). Story et al. investigated the association between social capital and antenatal care use in 10,739 women in India, and found that social capital at the community level was associated with all three types of antenatal care use (antenatal care use, skilled birth attendance, and complete child immunization). Interestingly, they found that social capital components with heterogeneous bridging ties were positively associated with all healthcare use, whereas components with strong bonding ties were negatively associated with the use of preventive care. Thus, in women with infertility symptoms, low bridging social capital may negatively affect the tendency to seek medical care and reduce the likelihood of receiving treatment or examinations, resulting in markedly lower ART use.

Second, young women with high social capital may experience substantial social pressure from other people, such as their partners or parents-in-law, to have a child (Balbo & Mills, 2011; Firouzbakht et al., 2020). Social networks, social pressure, and social capital can affect fertility decisions, particularly if there is little institutional support (Balbo & Mills, 2011). In highly homophilous communities, bonding social capital imposes a high economic and psychologic burden (Kawachi & Berkman, 2014). Women living with high social capital may feel social pressure to have a child, which may prompt them to undergo infertility treatment, resulting in higher ART use. The social context is highly dependent on culture. Cultures characterized by strong group binding and mutual obligation are typically found in Asian countries, including Japan; individualistic cultures in which individuals are independent are more typical of Western countries (Oyserman et al., 2002). Thus, social capital may differently affect ART use in different cultures.

ART is very costly; thus, affordability is considered a central factor that affects access to ART (Chambers et al., 2013; Dyer et al., 2020; Kelley et al., 2019; Smith et al., 2011). Using an international dataset, Chambers et al. investigated the effect of consumer cost on ART use. They identified an independent association between ART use and affordability: a decrease in the cost of a single ART cycle of 1 percentage point of disposable income was associated with a 3.2% increase in ART use (defined as the number of fresh non-donor cycles per million women of reproductive age) (Chambers et al., 2014). Their results suggest that household income affects affordability and ART use.

Interestingly, we did not find that prefectural-level additional subsidies were associated with ART use. People usually become familiar with the procedure of applying for government subsidies through repeated applications; however, it may be that application information does not reach young couples in Japan who may be planning to undergo ART. In the Japanese subsidy system, individuals must be patients themselves to be assessed for subsidy eligibility; therefore, young couples unfamiliar with the subsidy system may not apply. Thus, adequate information about additional subsidies from prefectures could help to promote ART use.

Similarly, we found no significant association between Gini coefficient and ART use. Studies have demonstrated that income inequality is associated with various health outcomes, including mortality and poor self-rated health; improving such inequality has recently received attention from governments (Centers for Disease Control and Prevention, 2013; Marmot et al., 2010; Marmot et al., 2012). High income inequality can lead to some segments of the population becoming impoverished and can hinder social cohesion (Subramanian & Kawachi, 2004), which may reduce ART use. One possible reason for the non-significant association between Gini coefficient and ART use is the threshold effect of income inequality (Kondo, 2012). Studies have reported a notable effect of income inequality in countries where such inequality is high (e.g., the United States and Britain); however, no such associations have been observed in countries with relatively low inequality (Nakaya & Dorling, 2005; Ross et al., 2000). Thus, the association between income inequality and ART use in different countries requires further investigation.

The present study is the first to empirically demonstrate an association between prefectural-level social capital and ART use using a cross-sectional survey and aggregated administrative data. However, this study has several limitations. First, we investigated women aged under 35 years who received government subsidies; the analysis excluded women who had not applied for any government subsidies. Because the subsidies are subject to an upper overall household income limit (<7.3 million yen [64,000 USD]), richer women were presumably excluded from the analysis and our findings may not accurately reflect the total number of women of that age undergoing ART in Japan. However, the analysis of individual data from Saitama Prefectural Government showed that almost the entire distribution was covered by the upper limit of overall household income (Fig. 3). Thus, we consider the use rate we applied to be valid.

Second, this study used prefectural-level aggregated data, not individual data. Accordingly, it was not possible to evaluate the effects of individual-level factors (such as individual socioeconomic status) and cross-level interactions between prefectural- and individual-level factors using a multilevel approach. Further, although we used multiple indicators to evaluate different aspects of social capital, there are other ways to measure the existence of community networks, such as perceived civic engagement and perceived trust (Reininger et al., 2013; Snelgrove et al., 2009), and other measures of social capital such as religious affiliation (Maselko et al., 2011), ethnicity (Johnson-Singh et al., 2018), employment (Sidorchuk et al., 2017), and online interaction (Rykov et al., 2020).

Third, because this was a cross-sectional study, the findings cannot be used to infer causality. There is also a possibility of ecological fallacy.

Fourth, owing to different funding systems and different compositions of ethnicity or race and cultural norms, our findings may not be generalizable to other countries or populations. Further, unmeasured factors, such as indicators for gender equality at the prefectural level (Chambers & Fauser, 2021) and ART tourism across prefectures, may have exerted effects. However, ART accessibility in Japan is the highest in the world (Dyer et al., 2020), so we assume that ART tourism across prefectures is rare. Therefore, to confirm our findings, further research using multilevel analysis is required to investigate pathways underlying the association between specific types of social capital and ART use.

Our results indicate that in addition to increasing financial accessibility, it may be possible to boost social capital at the prefectural level to increase ART use among young couples. Universal coverage of ART in Quebec has been widely reported (Tulandi et al., 2013); its introduction was effective in increasing ART use and substantially reducing multiple pregnancies and subsequent healthcare costs (Velez et al., 2014). However, in 2015, the system was discontinued owing to the increased cost to the healthcare system (Wei et al., 2021). Thus, it is necessary to consider increasing ART use from the perspective of sustainable healthcare. Additionally, social capital may have a negative effect on health (Villalonga-Olives & Kawachi, 2017). Therefore, further research on the association between social capital and ART use is essential before leveraging the concept of social capital to improve health interventions.

In conclusion, this cross-sectional survey using aggregated administrative data is the first to demonstrate an association between social capital and ART use. Although these data cannot be used to infer causal relationships, our findings suggest that in addition to improving financial access, an alternative approach of enhancing social capital could improve ART use. To confirm our findings, further research using multilevel analysis is required to investigate the association between specific types of social capital and ART use.

## Availability of data

Data are available upon reasonable request.

## Funding

This study was supported by a Health and Labor Sciences Research Grant (H30-Sukoyaka-Ippan-002) and the Japan Society for the Promotion of Science: KAKENHI (Grant Number 21H03193).

## Ethical statement

This study was approved by the institutional review board of Akita University (approval number, 1981; June 2018) and Saitama Medical University (approval number, 904; September 2019).

## CRediT authorship contribution statement

**Seung Chik Jwa:** Conceptualization, Formal analysis, Investigation, Writing – original draft. **Osamu Ishihara:** Writing – review & editing, Supervision, Visualization, Funding acquisition. **Akira Kuwahara:** Writing – review & editing, Validation. **Kazuki Saito:** Writing – review & editing, Funding acquisition. **Hidekazu Saito:** Writing – review & editing, Supervision. **Yukihiro Terada:** Writing – review & editing, Supervision. **Yasuki Kobayashi:** Conceptualization, Formal analysis, Methodology, Writing – review & editing. **Eri Maeda:** Data curation, Writing – review & editing, Funding acquisition, Visualization, Project administration.

## Declaration of competing interest

Dr. Osamu Ishihara has received an honorarium from Ferring Pharmaceuticals.
